# Application of three-dimensional computed tomography imaging and reconstructive techniques in lung surgery: A mini-review

**DOI:** 10.3389/fsurg.2022.1079857

**Published:** 2022-12-26

**Authors:** Mats T. Vervoorn, Maaike Wulfse, Firdaus A. A. Mohamed Hoesein, Margriet Stellingwerf, Niels P. van der Kaaij, Linda M. de Heer

**Affiliations:** ^1^ Department of Cardiothoracic Surgery, Division of Heart & Lungs, University Medical Center Utrecht, Utrecht, Netherlands; ^2^ Department of Radiology, Division of Imaging & Oncology, University Medical Center Utrecht, Utrecht, Netherlands; ^3^ Department of Pulmonology, Division of Heart & Lungs, University Medical Center Utrecht, Utrecht, Netherlands

**Keywords:** 3D reconstruction, lung surgery, computed tomogaphy, pulmonary surgery, VATS (video-assisted thoracic surgery), review, segmentectomy, lobectomy

## Abstract

**Background:**

Pulmonary surgery is an innovative discipline with increasing demands for minimally invasive techniques in complicated anatomical resections, warranting adequate preoperative imaging of relevant surgical anatomy to ensure safe and radical resection of target lesions. Over the recent years, the emergence of imaging techniques enabling three-dimensional reconstruction has exerted promising influence on pulmonary surgery, facilitating optimal surgical planning and easier identification of the spatial relationship between bronchovascular structures in the individual patient and aiding the safe resection of target pulmonary lesions. The goal of this mini-review is to provide an overview of three-dimensional computed tomography imaging within pulmonary surgery.

**Methods:**

The authors performed a targeted qualitative review of the literature to identify current trends and to provide better understanding of three-dimensional reconstruction within the boundaries of pulmonary surgery.

**Results:**

Three-dimensional reconstructive techniques can be used for resectability assessment, identification of surgically relevant interindividual anatomic variance and may improve perioperative outcomes.

**Discussion:**

Three-dimensional reconstruction using computed tomography imaging improves surgical planning and there is evidence that it results in shorter operative times, less intraoperative blood loss and lower rates of surgical conversion, as it can be applied both pre- and intraoperatively.

## Introduction

Over the last decades, the surge of minimally invasive techniques has impacted pulmonary surgery, accelerating the development of less invasive approaches for anatomical resections, primarily for early detected cancerous lesions (1–3). With the introduction of video-assisted thoracoscopic surgery (VATS) and evidence of its benefits compared to conventional thoracotomy (4, 5), the era of minimally invasive pulmonary surgery took off. Meanwhile, interest in increasingly smaller resections emerged, progressing from complete pneumonectomy to lobectomy and subsequent anatomic sublobar resections, such as segmentectomy. These procedures are associated with lower complication rates and increased residual pulmonary function, while maintaining adequate surgical radicality in early stage lung cancer as indicated by similar progression-free survival and local recurrence rates (6–11).

With emerging evidence of the benefits of increasingly smaller resections through minimally invasive approaches, the added potential complexity of these procedures poses an important challenge. They require precise knowledge of the individual patient anatomy regarding bronchovascular structures, while taking the effects of pulmonary deflation during surgery into account. This is especially true for segmental resections because of the individual variability at segmental levels. Innovations in imaging techniques might prove valuable in overcoming these challenges, as technological improvements regarding imaging modalities have provided surgeons with additional ways to prepare for individual cases. Mainly computed tomography (CT) based technologies offer three-dimensional (3D) reconstruction that creates many options for pre- and intraoperative planning that were previously unavailable. Compared to conventional 2D-imaging, they offer the advantage of improved spatial orientation and identification of surgically relevant structures and their interindividual variance.

In this mini-review, we provide an overview of the clinical application of these techniques in the context of pulmonary surgery. We will not focus on the specific techniques for 3D reconstruction, such as volume rendering and maximum intensity projection, but describe the added clinical value of such techniques in general.

## Materials and methods

**Figure 1:**
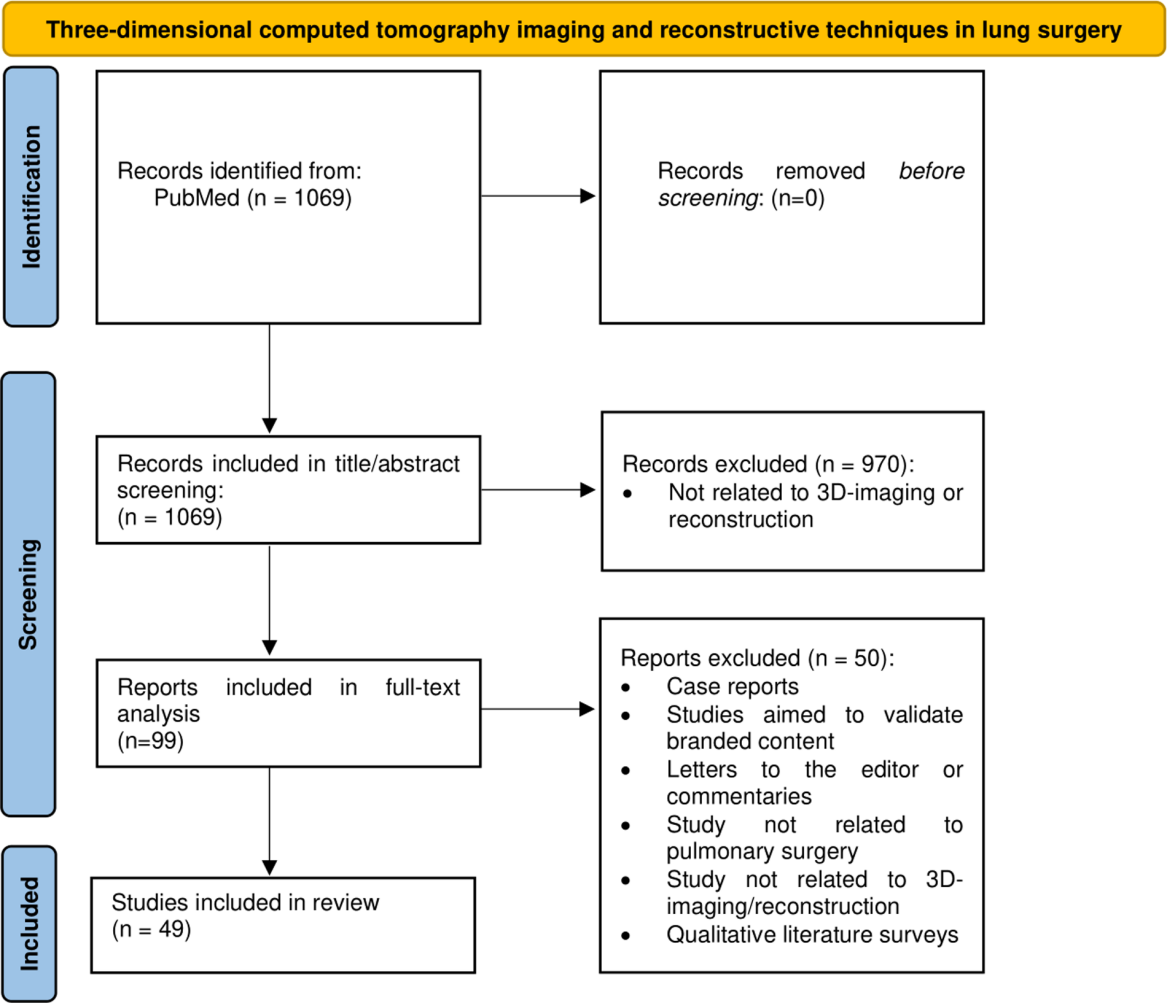
Flowchart of the conducted search. 3D: three-dimensional.

The authors (MV, LH) performed a targeted qualitative review of the literature within the PubMed database (date: 25th of October 2022) to identify current trends and to provide better understanding of 3D reconstruction within the boundaries of pulmonary surgery. Key search words included terms related to 3D reconstruction and different surgical approaches for pulmonary surgery, including VATS, pneumonectomy, lobectomy, segmentectomy, etc. (Appendix 1). The search string is highlighted in Figure 1.

## Results

### Resectability assessment

Early-stage lung cancer is an important indication for pulmonary resection. To achieve adequate resection of a cancerous lesion, it is crucial that sufficient resection margins are respected. Multiple studies demonstrated that both lobectomy and segmentectomy are able to provide adequate resection margins, although the chance of local recurrence is increased in cases of segmentectomy with a margin to tumor size ratio <1 cm or if tumor size exceeded 2 cm (2, 3, 12, 13). In preoperative planning, securing an adequate resection margin is an important consideration when deciding on the operative approach and surgical planning. In case of segmentectomy, it is critical to identify the intersegmental veins as the boundary lines of the pulmonary segments and to identify the target segmental bronchi as the vertical surgical margin.

To date, many surgeons use traditional 2D CT imaging to estimate the resectability of a lesion and decide on operative approach. However, 3D-imaging using CT-based simulation techniques, sometimes assisted by artificial intelligence (14), can be used to accurately identify individual segments by tracing bronchovascular anatomy (15, 16), making it possible to estimate the probability that a cancerous lesion in a single segment has safe anatomical margins for segmentectomy, or needs change of the original surgical plan to another resection (for instance: bi-segmentectomy, lobectomy) if surgical margins are deemed insufficient (17–21). Comparative studies support this by describing a lower percentage of inadequate resection margins when preoperative assessment was conducted using 3D reconstruction instead of conventional 2D-imaging (22, 23). Evidence by Bakhuis et al. (24) supports this. They describe their experience with 3D reconstructions for preoperative planning in 50 patients with an indication for pulmonary segmentectomy and reported an adjustment of the preoperative plan that ensured radical resection in 52% of patients based on the acquired 3D reconstructions. They also found that in 14% of patients the tumor was localized in a different segment than assessed by conventional 2D-imaging. Nakamoto et al. (25) demonstrated that it was also possible to identify candidates for smaller, so-called “superselective” segmentectomy for diagnostic resection of small nodules located at a depth greater than 20 mm from the pleural surface. Using 3D-reconstruction, they were able to adequately identifying surgical planes based on arterial branching patterns up to the fifth-order that still satisfied the requirements for sufficient surgical margin in indeterminate small and deep pulmonary nodules, resulting in low volumes of resected parenchyma and thus preservation of pulmonary function.

The clinical efficacy and safety of preoperative 3D reconstruction in assessing resectability prior to anatomical sublobar resection is demonstrated by a recent study conducted by Hamada et al. (26) They showed a total 5-year overall survival, lung cancer-specific overall survival, and recurrence-free survival rates after VATS sublobar resection based on preoperative 3D reconstruction for surgical planning of 92.5%, 100% and 98.2% respectively in patients with stage IA non-small cell lung cancer. These results suggest that sublobar resection under 3D-reconstructive guidance achieves sufficient surgical margin for satisfactory long-term outcome.

Additionally, preoperative knowledge regarding the histologic origin of a specific tumor, if available, may also influence surgical decision-making. In most instances, preoperative histological specimens are required to tissue-type a tumor and decide on the most fitting surgical resection. In this regard, 3D-reconstructive techniques might also be of added value. A recent study used 3D reconstruction to identify hallmarks suggestive of malignancy, such as the presence of a solid component, lobulation, vascular convergence, or an air cavity density, as well as nodule size (27). These results highlight the potential benefit of preoperative 3D reconstruction in estimating the origin of ground glass nodules, thereby facilitating adequate surgical planning.

### Identification of anatomical landmarks and interindividual variance

Pulmonary bronchovascular patterns are diverse and constitute complex spatial configuration. Surgeons must be familiar with both the general and patient-specific anatomy of pulmonary bronchovascular structures to conduct anatomical (sublobar) resections safely and adequately. Multiple studies showed that by using preoperative 3D reconstruction it was possible to accurately identify up to 98% of surgically relevant and potentially complicating pulmonary artery branches, including anomalous or uncommon variations in vascular branching patterns. Thereby, 3D reconstruction facilitates surgical resections by avoiding blind dissection of vessels and increases surgeons confidence in their knowledge of the spatial relationship between the lesion and adjacent anatomical structures in the individual patient (18, 19, 28–43).

### Perioperative outcome

Adequate resectability assessment and enhanced identification of potentially complicating anatomical variants suggest improved perioperative outcome when 3D-reconstructive techniques are used in the preoperative work-up. Results from multiple, primarily retrospective, comparative studies in patients operated on for a variety of pulmonary diseases, confirm this hypothesis, although the quality of evidence is debatable due to their retrospective nature (Table 1). In these studies, 3D-imaging is associated with shorter operating times (22, 36, 37, 44–46), more extensive mediastinal lymph node dissection (23, 36), a reduction in stapler reloads (44), reduced air loss on postoperative days 1–3 (44) and decreased intraoperative blood loss (36). No significant difference in other surgery-related parameters was found, such as length of hospital-stay, overall recovery, incidence of other postoperative complications and residual pulmonary function (22, 23, 36, 44, 45). Furthermore, there is evidence from multiple studies that preoperative 3D-imaging aided in selection of the appropriate surgical entry site tailored to the individual patient by taking their specific anatomy into account, resulting in less perioperative switching in surgical approach and lower conversion rates (36, 47, 48).

**Table 1:** Summary of the results from comparative studies regarding perioperative outcome. PSM: propensity score-matched; GGO: ground glass opacity; BMI: body mass index; FEV1: forced expirational volume in 1 s; 3D: three-dimensional; CT: computed tomography; POD: postoperative day; (U)VATS: (uniportal) video-assisted thoracoscopic surgery; MDCT: multidetector CT; RR: risk ratio.

Author	Year	Study design	Imaging modality	Outcome
Wang (44)	2022	Retrospective analysis of 97 patients undergoing complex segmentectomy of the lower lobe.	Preoperative 3D reconstruction vs. conventional 2D imaging.	Significantly reduced operative time (111.4 vs. 127 min; *P* = 0.007), required stapler reloads (9.0 vs. 10.4; *P* = 0.009) and air leakage on postoperative days 1-3 (11.9 vs. 10.4; *P* = 0.027).
Wu (23)	2021	Retrospective PSM-analysis comparing precise segmentectomy to routine segmentectomy for patients with single or multiple GGO's as uniportal procedures.	Preoperative 3D-reconstruction and real-time surgical guidance using contrast-enhanced CT-imaging (*n* = 55) vs. serial CT-imaging (*n* = 55).	Increased operating time (74 vs. 55 min; *P* < 0.01), increased number of dissected lymph nodes (5 vs. 3; *P* < 0.01), increased air leakage on POD1 (56.4% vs. 16.4%; *P* = 0.020) in 3D group.
				Trend towards decreased amount of cases with inadequate resection margins (0 vs. 4; *P* = 0.12) in 3D group.
		PSM factors were sex, BMI, surgery type, % FEV1.		
Zhu (36)	2021	Retrospective analysis of 198 consecutive cases undergoing curative UVATS lobectomy with 1:1 PSM. PSM factors were age, sex, BMI, maximal lesion size, type of surgical resection, and lymph node dissection.	Preoperative 3D-reconstruction using 64-slice MDCT for pulmonary arteriography/venography (*n* = 53) vs. conventional 2D MDCT (*n* = 53).	Shorter operative time (145.7 *vs.* 164.2 min; *P* = 0.014), increased number of dissected lymph nodes (8.19 *vs.* 5.78; *P* = 0.024), reduced intraoperative blood loss (60.4 *vs.* 100.8; *P* = 0.009) in 3D group.
Qiu (45)	2020	Retrospective analysis of 298 cases that underwent anatomical partial lobectomy.	Preoperative 3D-reconstruction using CT-imaging vs. 3D-printing vs. conventional 2D CT-imaging.	Shorter operative time using 3D-imaging (116.1 min; *P* = 0.04) and 3D-printing (99.6 min; *P* < 0.001) compared to conventional 2D-CT imaging (125.1 min) for complex segmentectomy, decreased perioperative blood loss using 3D-printing (12.9) compared to 3D-reconstruction (20.9 ml; *P* = 0.001) and conventional 2D-CT (18.2; *P* = 0.02).
Xue (22)	2018	Retrospective analysis of 68 cases of GGO ≤2 cm, diagnosed as cT1aN0M0 lung cancer, undergoing VATS segmentectomy.	Preoperative 3D-reconstruction simulation of CT images (*n* = 36) vs. conventional 2D CT images (*n* = 32).	Shorter operating time (111 min vs. 139 min; *P* = 0.03) and lower amount of cases with inadequate resection margin (0 vs. 4; *P* = 0.04) in 3D group. Trend towards decreased intraoperative bleeding (129.1 vs. 106.4 ml; *P* = 0.06) in 3D group.
Hagiwara (37)	2014	Retrospective analysis of 179 consecutive cases of VATS anatomical lung resection.	Preoperative 3D-reconstruction using 64-channel MDCT imaging (*n* = 124) vs. conventional 2D MDCT (*n* = 55).	Increased risk for extended operative times (RR 2.3; *P* = 0.021) and higher risk for operative complications (RR 2.9; *P* = 0.074) in the 2D group

When comparing 3D-imaging to more other reconstructive techniques, such as 3D-printing, there is evidence that the use of 3D-printing has additional benefits regarding reduction of operative time, perioperative blood loss and conversion rates when compared to both 3D-imaging and conventional 2D-imaging, especially in more complex cases (45, 48).

### Intraoperative application

Besides its role in preoperative assessment, the use of 3D-reconstructive techniques during surgery has also been reported. Examples include the use of a tablet during surgery to display preoperatively constructed 3D images, with specific focus on pulmonary bronchovascular anatomy related to the target lesion. Several authors report that the reconstructions displayed on the tablet helped to identify important anatomic structures and their spatial relationship, while being able to review and manipulate them during surgery. This significantly benefitted their intraoperative understanding of the relevant surgical anatomy and enhanced their ability to safely perform anatomical resections (49, 50).

Another example includes binocular stereo-navigation using 3D polarized glasses which displayed 3D-reconstructed images during surgery, to be used as an intraoperative navigation tool during VATS. This enabled easier and accurate identification of relevant bronchovascular structures, while achieving sufficient resection margins (51).

Chen et al. (48) used 3D-printed models to effectively aid surgeons in intraoperative identification of relevant surgical landmarks and concluded that 3D-printed pulmonary models enable easier localization of target lesions, selection of important anatomical landmarks as reference points and identification anatomical variations during the procedure from multiple angles. Qiu et al. (45) found that 3D-printed models displayed during surgery reduced perioperative blood loss by preventing vascular trauma in patients undergoing VATS anatomical partial-lobectomy for stage I lung cancer.

### Educational value

The high-quality 3D reconstructions rendered by modern CT-imaging techniques might also possess educational value in obtaining informed consent from patients regarding the surgical procedure they are subjected to. Also, evidence suggests it might improve surgical residency training (37, 45, 52, 53). The latter was investigated by Zhang et al. (46), who found evidence of faster learning of complex VATS procedures in surgical residents that used 3D reconstructions in their surgical preparation, compared to older surgeons that did not have these sophisticated techniques at their disposal, although age might have confounded these results. There was also a tendency for shorter operating times when preoperative resection simulation was applied using 3D-reconstructed anatomical models.

## Discussion

Pulmonary surgery is an innovative discipline with increasing demands for minimally invasive techniques in complicated anatomical resections. Proper, usually CT-based, imaging to assess surgical anatomy in the individual patient during preoperative assessment is an important step in the preoperative work-up towards surgery. Therefore, sophisticated 3D-reconstructive techniques might positively influence individual patient outcomes. The accumulating evidence in favor of using 3D reconstruction over conventional 2D-imaging is expanding, with recent studies providing evidence of shorter operating time, more extensive lymph node dissection, reduced perioperative blood loss and lower conversion rates when using advanced 3D-reconstructive techniques both pre- and intraoperatively.

Furthermore, it has been suggested that these techniques might also aid in estimating the chance of malignancy by identifying certain hallmarks of a suspicious nodule by 3D reconstruction, thereby improving preoperative planning and potentially reducing intraoperative uncertainty regarding the specific origin of a nodule, which is now often solved by either time-consuming frozen section sampling or surgical resection, which might prove redundant when postoperative histological analysis shows a benign lesion.

Despite the limited comparative evidence, the perceived added value of 3D reconstruction is subjectively highlighted by most authors. In their opinion, 3D reconstruction resulted in a better preoperative understanding of relevant individual surgical anatomy and variations, which enabled accurate surgical planning and improved the ability to identify segmental and subsegmental borders. Furthermore, it prevented accidental injury to bronchovascular structures, improved intraoperative navigation, patient counseling and surgical training, it reduced the unnecessary exploration of surrounding structures during surgery and enabled better prediction of patients at risk of surgical conversion (22, 23, 36, 45, 48, 54). When Qiu et al. asked surgeons contributing to their study about the perceived benefits regarding 3D-imaging compared to conventional 2D-techniques, 88% of them agreed that 3D models provided a better understanding of the thoracic segmental anatomy and that they were useful for improving communication with patients and colleagues, while 81% of surgeons agreed or strongly agreed that 3D-modeling might help to diminish potential surgical complications.

An often-mentioned limitation to the use of 3D reconstruction in surgical planning is the added time constraints regarding processing time for visualizing relevant structures. However, most studies report that with recent advances in reconstructive software, the processing times have been reduced to minutes, rendering this argument invalid (37, 52, 55–58). Regarding the use of 3D-printing, costs might be a limiting factor. However, Smelt et al. (39) reported that the costs of production of single print reconstructions are similar to the cost of a single stapler reload often used in thoracic surgery and they identified the costs of the printer itself as the major financial burden. In current times, major centers often have access to a 3D-printer, which might diminish associated costs.

An important limitation to this review is related to the retrospective nature of most comparative studies regarding 3D reconstruction vs. conventional 2D-imaging, which makes it difficult to draw definitive conclusions regarding patient outcome due to the inherently increased risk of bias in these studies. An encouraging finding is the confirmation of the beneficial outcomes related to the application of 3D-imaging and reconstructive techniques, such as 3D-printing in a single prospective study (48). Nevertheless, one should be cautious with interpretation of these results and definitive recommendations should be based on future prospective and comparative studies.

Regarding future research, we propose the next step in the validation of 3D-imaging in pulmonary surgery would be the conductance of a prospective, randomized-controlled trial for direct comparison of conventional 2D-imaging vs. 3D-reconstructive techniques to further elucidate the clinical potential of 3D reconstruction in preoperative assessment and investigate its influence on patient outcomes. Another important finding requiring further investigation concerns the indication that 3D reconstruction of pulmonary nodules might be helpful in risk prediction of malignancy (27). This is supplemented by reports of such reconstructed images of pathological specimens after resection, that aided in establishing a definitive diagnosis for pulmonary nodules of unclear origin (59, 60). Because the specific oncological origin of a suspected tumor is of major importance in deciding on surgical approach and now requires invasive preoperative procedures for tissue typing, methods of 3D reconstruction that aid in elucidating the nature of a suspected lesion, perhaps in combination with emergent artificial-intelligence based algormight prove of added value.

In conclusion, evidence suggests a benefit of 3D reconstruction in pulmonary surgery by reducing operating times, intraoperative blood loss and lowering the rate of surgical conversion. It enables adequate surgical planning surgical, assessment of resection margins in oncological surgery and might be of added value in both residency training and patient education. By visualizing the complex spatial relationship between surgically relevant bronchovascular structures, it enables surgeons to perform minimally invasive and complex anatomical resections safely and adequately. Future research should focus on confirming these results in prospective clinical trials.

## APPENDIX 1. Search string

(three-dimensional imaging[Title/Abstract] OR three dimensional imaging[Title/Abstract] OR 3D imaging[Title/Abstract] OR 3-D imaging[Title/Abstract] OR 3-dimensional imaging[Title/Abstract] OR 3 dimensional imaging[Title/Abstract] OR 3D reconstruction[Title/Abstract] OR 3 dimensional reconstruction[Title/Abstract] OR three dimensional reconstruction[Title/Abstract]) AND (Pulmonary surgery OR lung surgery OR thoracic surgery OR video assisted thoracic surgery OR VATS OR lobectomy OR segmentectomy OR pneumonectomy OR pneumectomy OR bullectomy OR Thoracoscopic surgery OR wedge resection OR sleeve resection).
